# Two Decades of Active Surveillance for Prostate Cancer in a Single-Center Cohort: Favorable Outcomes after Transurethral Resection of the Prostate

**DOI:** 10.3390/cancers14020368

**Published:** 2022-01-12

**Authors:** Sarah Hagmann, Venkat Ramakrishnan, Alexander Tamalunas, Marc Hofmann, Moritz Vandenhirtz, Silvan Vollmer, Jsmea Hug, Philipp Niggli, Antonio Nocito, Rahel A. Kubik-Huch, Kurt Lehmann, Lukas John Hefermehl

**Affiliations:** 1 Department of Surgery, Division of Urology, Kantonsspital Baden, 5400 Baden, Switzerland; marc.hofmann@ksb.ch (M.H.); kurt.lehmann@ksb.ch (K.L.); lukas.hefermehl@ksb.ch (L.J.H.); 2 Division of Urology, Brigham and Women’s Hospital, Harvard Medical School, Boston, MA 02115, USA; venkatmr88@gmail.com; 3 Department of Urology, University Hospital, LMU Munich, 80539 Munich, Germany; alex.tamalunas@web.de; 4 Department of Mathematics, Swiss Federal Institute of Technology, 8092 Zurich, Switzerland; mvandenhi@student.ethz.ch (M.V.); svollmer@student.ethz.ch (S.V.); hugjs@student.ethz.ch (J.H.); pniggli@student.ethz.ch (P.N.); 5 Department of Surgery, Kantonsspital Baden, 5400 Baden, Switzerland; antonio.nocito@ksb.ch; 6 Department of Radiology, Kantonsspital Baden, 5400 Baden, Switzerland; rahel.kubik@ksb.ch

**Keywords:** active surveillance, prostate cancer, transurethral resection of the prostate, TURP, prostate cancer results

## Abstract

**Simple Summary:**

Prostate cancer is the most commonly diagnosed cancer in men. Active surveillance—repeated measurements of prostate specific antigen, digital rectal examination, and prostate biopsies—is an alternative treatment option to active therapy, such as radical prostatectomy or radiotherapy, for patients with low grade prostate cancer. It aims to reduce overtreatment and negative side effects of active treatment. However, long-term outcome data is rare. As we prospectively collected data since the beginning of active surveillance in our clinic in 1999, our study provides insights into long-term outcomes after two decades of active surveillance.

**Abstract:**

Objective: To report the outcomes of active surveillance (AS) for low-risk prostate cancer (PCa) in a single-center cohort. Patients and Methods: This is a prospective, single-center, observational study. The cohort included all patients who underwent AS for PCa between December 1999 and December 2020 at our institution. Follow-up appointments (FU) ended in February 2021. Results: A total of 413 men were enrolled in the study, and 391 had at least one FU. Of those who followed up, 267 had PCa diagnosed by transrectal ultrasound (TRUS)-guided biopsy (T1c: 68.3%), while 124 were diagnosed after transurethral resection of the prostate (TURP) (T1a/b: 31.7%). Median FU was 46 months (IQR 25–90). Cancer specific survival was 99.7% and overall survival was 92.3%. Median reclassification time was 11.2 years. After 20 years, 25% of patients were reclassified within 4.58 years, 6.6% opted to switch to watchful waiting, 4.1% died, 17.4% were lost to FU, and 46.8% remained on AS. Those diagnosed by TRUS had a significantly higher reclassification rate than those diagnosed by TURP (*p* < 0.0001). Men diagnosed by targeted MRI/TRUS fusion biopsy tended to have a higher reclassification probability than those diagnosed by conventional template biopsies (*p* = 0.083). Conclusions: Our single-center cohort spanning over two decades revealed that AS remains a safe option for low-risk PCa even in the long term. Approximately half of AS enrollees will eventually require definitive treatment due to disease progression. Men with incidental prostate cancer were significantly less likely to have disease progression.

## 1. Introduction

Prostate cancer (PCa) is the most commonly diagnosed cancer in men and third most common cause of cancer death [1]. Treatment options for patients with low-grade PCa include radical prostatectomy, radiotherapy, or active surveillance. There is no significant difference in prostate cancer-specific mortality among those treatments [2,3]. Active surveillance (AS) for localized, low-risk prostate cancer aims to reduce over-treatment and minimize the negative side effects of active treatment, such as incontinence, erectile dysfunction, strictures, and other lower urinary tract symptoms (LUTS), while retaining the option for curative treatment at the first sign of disease progression [4]. As such, patients follow up on a routine schedule with regular measurements of prostate specific antigen (PSA), digital rectal examinations (DRE), and prostate biopsies. Over the last decade, AS has joined the urologist’s armamentarium as a standard treatment option. However, few studies have reported outcomes beyond ten years of follow-up [5,6,7,8].

In 1999, our institution was one of the first European centers to enroll patients in a standardized AS protocol, noting that at that time, the standard of care was to offer curative treatment for those with low-risk disease diagnosed via a templated biopsy (T1c) as well as via transurethral resection of the prostate (TURP) (T1a/b). Though it was controversial at the time, we included men with T1a/b PCa in our cohort of over 410 AS patients [9]. Now, more than two decades later, we present our long-term outcomes and also attempt to ascertain that AS remains a safe option for PCa patients diagnosed via TURP. Since mpMRI (multi-parametric magnetic resonance imaging) has become increasingly important and widely implemented in the recent years, the role of mpMRI in AS remains unclear [10,11]. We would like to present our data on the role of mpMRI in AS.

## 2. Patients and Methods

Patients with low-risk PCa who initially opted for AS over other curative treatment options [12] were enrolled in the study between December 1999 and December 2020. Inclusion criteria included those with T1a/b disease as well as patients with (a) disease limited to one lobe of the prostate, with no more than two positive cores with less than 5 mm of tumor length each, (b) no evidence of Gleason grade disease >3, and (c) an equivalent or lower Gleason grade on a confirmation biopsy performed 3–6 months after initial diagnosis. In selected equivocal cases, we also enrolled those with clinically low-risk Gleason 7 disease (for instance, those with a single biopsy showing 1 mm of Gleason 3 + 4 disease).

We performed standardized TRUS-guided prostate biopsies (with six randomly distributed cores on each side) through 2014, at which point targeted MRI/TRUS fusion biopsies (Artemis, Eigen, CA, USA) were introduced at our institution. Thereafter, we mostly performed targeted biopsies and omitted the requirement for a confirmation biopsy [13]. TURP was performed in accordance with EAU guidelines [14].

Follow-up appointments (FU) were scheduled every 6 months. PSA testing and DREs were performed at each FU. In those without a PSA increase and a negative DRE, we performed biopsies yearly for the first six years and then every other year through the end of the study period. Indications for an earlier biopsy included (a) a PSA increase of ≥0.5 ng/mL from prior, (b) a newly discovered, suspicious lesion on DRE, and (c) suspicious lesions seen on mpMRI (since 2014). Reclassification was defined as the pathological progression of the tumor (Gleason score >7, bilateral disease, and/or >2 unilateral biopsies with >5 mm of disease per core). Those who were reclassified were offered curative treatment. Rising PSA alone did not serve as a trigger for intervention. To eliminate follow-up bias, we only included patients with at least one FU in our analysis. After reclassification, we collected data on the respective treatment the patient received, treatment failure (recurrence; need for second line treatment), and death.

The primary endpoint of this study was the reclassification rate. Secondary endpoints included overall survival (OS), cancer specific survival (CSS), and drop-out rate.

Descriptive statistics were used to characterize the cohort. Kaplan–Meier curves, log-rank tests and Cox regression models are part of the survival analysis. Kaplan–Meier estimation is a frequently used method to deal with differing times until a certain event has happened. In this study, an event can be reclassification, death, or recurrence. The log-rank test determines if there is no difference between patient groups in the probability of an event at any time point. In Cox regression models, the association between survival time and predictor variables such as PSA, age, or prostatic volume can be assessed. The statistical analysis is completed by a mixed-effects logistic regression. Here, we analyzed the reclassification probability by including additional information obtained in the follow-ups such as regularity of follow-up attendance or PSA at follow-up. Data analysis was approved by the local ethics authorities.

## 3. Results

### 3.1. Overall Study Metrics

Between December 1999 and December 2020, a total of 413 patients with a median age of 67 years (IQR 62–71) were included in our AS protocol. Of these, 391 patients followed-up at least once and qualified for further analysis. 183 patients (46.8%) remained on AS at the time of our analysis. 124 patients (31.7%) were diagnosed by TURP and 267 (68.3%) were diagnosed by TRUS biopsy, of which 87 (32.6%) were diagnosed using modern targeted MRI/TRUS fusion biopsy techniques (Table 1). Of the anticipated 4086 PSA measurements and 1020 biopsies across all subjects during the total study period, 3361 (82%) and 524 (51%) were performed, respectively. The median PSA doubling time was 49 months, while the median PSA density (PSAD) at enrollment was 0.11 ng/mL/cm^3^ (IQR 0.08–0.16). Interestingly, the PSAD in patients with T1a/b disease was significantly lower than those with T1c disease, at 0.08 ng/mL/cm^3^ (IQR 0.04–0.1) versus 0.12 ng/mL/cm^3^ (IQR 0.08–0.17), respectively (*p* = 0.0004) (Table 2).

The median reclassification time was 11.2 years (95% CI [10; N/A]), while 25% of patients were reclassified within 4.58 years (95% CI [3.78; 6.48]). 6.6% (*n* = 26) of patients opted to switch to watchful waiting (WW). The loss to FU (LTF) rate was 17.4% (*n* = 68), and LTF occurred after a median 39.75 months (IQR 26.71–56.54) at a median age of 70.25 years (IQR 65.67–75.04) (Table 3). The median LTF time was measured as the time between initial diagnosis/AS enrollment and the last known FU date. Of the 68 lost to FU, 13 (19.1%) changed providers and no longer followed up at our institution, 9 others (13.2%) failed to present to two consecutive FUs and were ultimately considered LTF, 24 (35.3%) actively discontinued further FU examinations and, to our knowledge, did not pursue definitive therapy, and the remaining 22 (31.4%) were LTF for unknown reasons.

**Figure 1:**
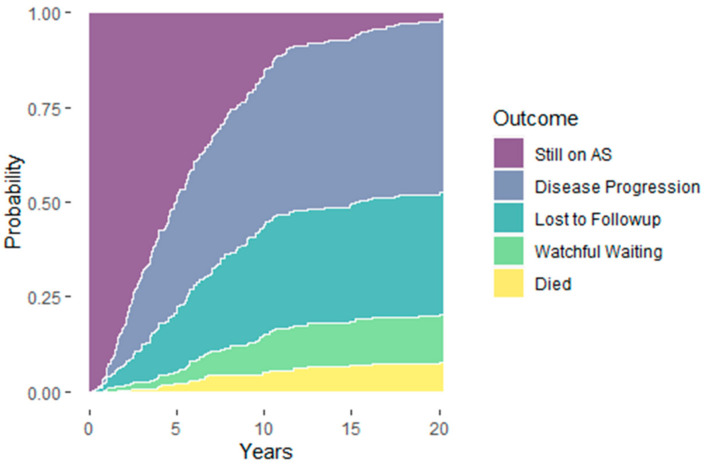
Probability of staying in active surveillance and probability of discontinuing active surveillance.

The probabilities of remaining on AS at 5, 10, 15, and 20 years after study enrollment was 49.6, 16.4, 6.9, and 2.4%, respectively, while the probabilities of disease progression (29.4, 39.8, 44.0, and 45.7%), conversion to WW (3.2, 10.2, 11.6, and 12.5%), loss to FU (15.9, 28.8, 30.6, and 32.2%), and all-cause mortality (2.0, 4.9, 6.9, and 7.1%) all expectedly increased over the same period of time (Figure 1). The median time to curative treatment was 2.55 years after initial diagnosis and AS enrollment (IQR 1.61–5.5). Table 4 summarizes the curative treatment modalities pursued after disease reclassification. Interestingly, of the 101 patients who ultimately pursued active treatment due to progression, only 9.4% demonstrated PCa recurrence after first-line treatment and needed second-line therapy, while 4 men (1%) showed metastatic disease.

**Figure 2:**
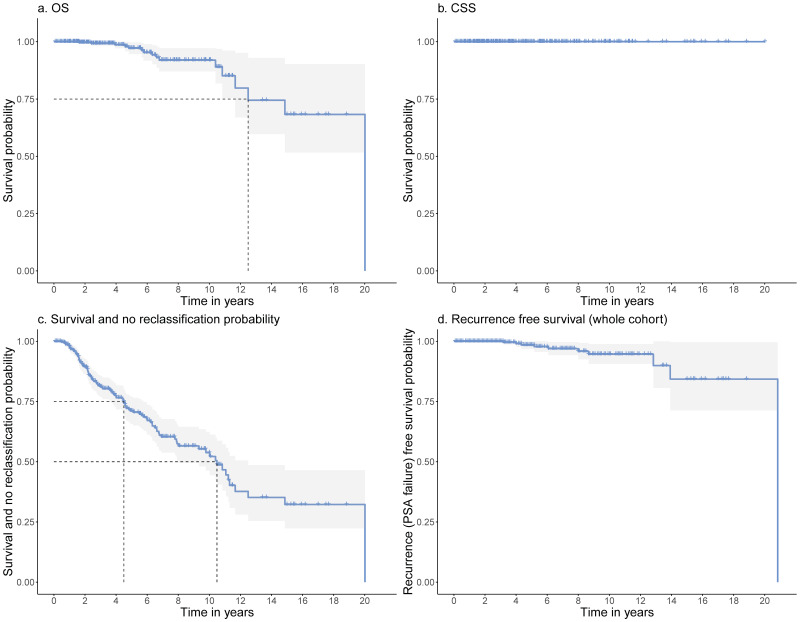
(**a**) OS at 92.3%, the dashed line shows the first quartile of 12.5 years; (**b**) CSS at 99.7%; (**c**) Survival and no reclassification probability, the dashed lines show the first quartile (4.5y) and median (10.5y); (**d**) Recurrence free survival rate at 89.8% (whole cohort). CI in light grey.

By the conclusion of the study, 16 patients (4.1%) died, and only one of these deaths (1/394 = 0.3%) was directly attributed to PCa itself, thereby rendering the CSS at 99.7% and OS at 92.3%. The recurrence-free survival (RFS) rate was 89.8%. First quartile of OS according to KM is 12.5 years with a lower bound of 11.7 of the corresponding 95% confidence interval, meaning 25% of the patients die in the first 12.5 years (Figure 2). The median overall progression rate was 24.3% (*n* = 95).

### 3.2. Impact of TURP vs. Biopsy on Reclassification Rates

**Figure 3:**
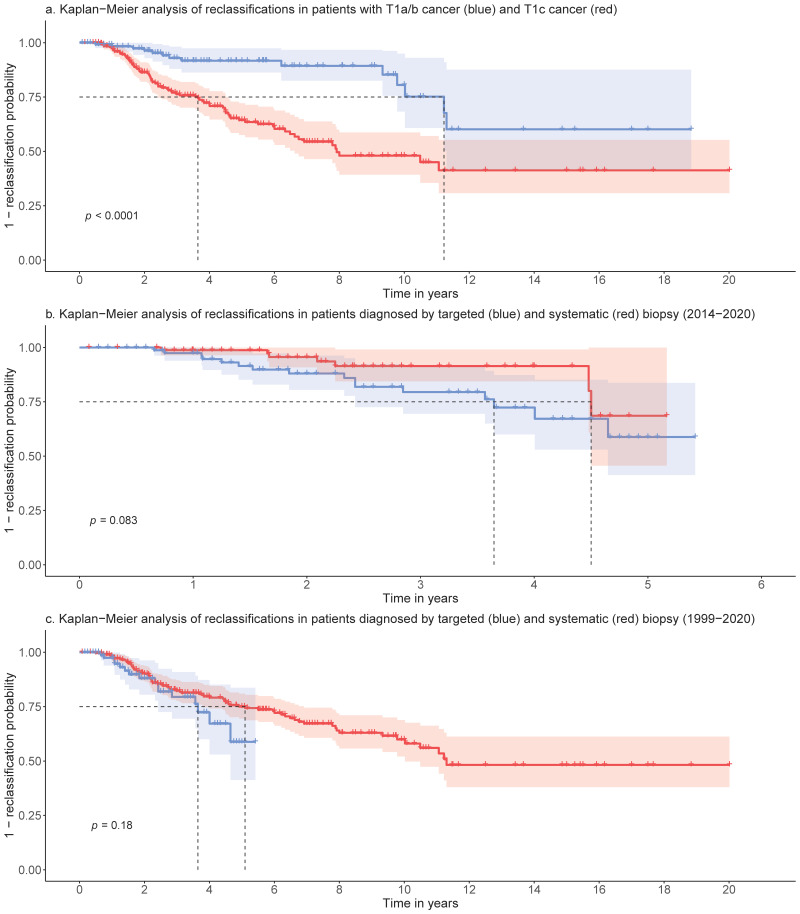
(**a**): Kaplan–Meier analysis of reclassifications in patients with T1a/b cancer (blue) and T1c cancer (red): 25% of the patients are reclassified within 3.6y (T1c) and 11.2y (T1a/b). A total of 95% CI in light blue and light red, (**b**): Kaplan–Meier analysis of reclassifications in patients diagnosed by targeted (blue) and systematic (red) biopsy using the data from 2014–2020: 25% of the patients are reclassified within 3.6y (targeted) and 4.5y (systematic) (**c**): Kaplan–Meier analysis of reclassifications in patients diagnosed by targeted (blue) and systematic (red) biopsy using the data from 1999–2020.

We next compared the reclassification rates between patients incidentally diagnosed with PCa on TURP (T1a/b) and those diagnosed by a templated biopsy (T1c). A total of 25% of the patients in the T1c group were reclassified within 3.6 years of their initial diagnosis (95% CI [2.42, 4.58]), while 25% of those in the T1a/b group were reclassified significantly later at 11.2 years (95% CI [9.78, N/A]; *p* < 0.0001) (Figure 3a).

### 3.3. Impact of Biopsy Modality on Reclassification Rates

Furthermore, we compared the reclassification rates between patients diagnosed using both biopsy modalities–the systematic TRUS biopsy, where 6 systematic samples of each side of the prostate are extracted and the targeted MRI/TRUS fusion biopsy, where targeted biopsies out of suspect lesions on the mpMRI are extracted, additionally to the systematic samples. Ultimately, our analysis revealed no significant differences between the groups (Figure 3b,c) using data from all patients included since the introduction of targeted MRI/TRUS fusion biopsies at our institution between 2014 and 2015 (*n* = 87, *p* = 0.083), and also using the data of the entire cohort since 1999 (*n* = 394, *p* = 0.18). A total of 25% of the patients in the fusion biopsy group were reclassified within 3.6 years of initial diagnosis (95% CI [2.42, N/A]), whereas 25% of those who received standard TRUS biopsies were reclassified within 4.5 years (95% CI [4.48, N/A]).

### 3.4. Predicting Reclassification

We lastly documented and analyzed factors at enrollment (such as age, T1c status, PSA, prostate volume, and PSAD) that might have contributed to higher reclassification rates using Cox regression modelling. Reclassification was significantly lower in those with a negative “T1c-status” (*p* = 0.009) and higher prostate volumes (*p* = 0.005). The higher the prostate volume, the lower the risk of PCa progression and, ultimately, reclassification. The patient’s age and PSAD bore no significant impact on the hazard of reclassification.

Next, we performed a mixed-effects logistic regression analysis featuring the variables “regular FU” (regular = if the patients adhered strictly to the protocol, irregular = otherwise), “PSA at FU”, “PSA change” (the difference in PSAs between two consecutive FU periods), “DRE”, “Gleason score”, “biopsy”, and “number of FUs”). Only “regular FU” (*p* = 0.01), “biopsy” (*p* = 0.001), and “PSA at FU” (*p* = 0.05) were factors that significantly increased the risk of reclassification within the following 6–12 months. PSA changes in patients with T1a/b disease conferred a significantly higher risk of reclassification than similar changes in their T1c counterparts (*p* = 0.001).

## 4. Discussion

Conducted in a real-world, single-center setting and with a duration of over 20 years, our study demonstrates the long-term outcomes of a unique cohort of over 390 men with low-risk PCa managed with AS. With an OS of 92.3% and CSS of 99.7% in our cohort, AS is a safe, long-term treatment option for those with low-risk disease. Only one patient in this cohort of 413 patients has died of prostate cancer, explaining the high CSS. In this particular case, in the early stages of the cohort, the patient might have been under-staged at the time of inclusion, as he showed a Gleason score 10 in the first follow-up biopsy in the pre-MRI era.

Our results reiterate findings from other studies that were conducted over shorter durations and at least five years after our study had begun, including PRIAS (2006), HAROW (2008–2013), and The Movember Foundation’s GAP3 cohort (2014–2016; a database to which we have also contributed) [15,16,17,18,19,20,21].

Here, we found that (a) the likelihood of remaining on AS for 20 years is below 3%, with a less than 50% chance of progression in that time, (b) the probability of LTF goes up the longer someone remains enrolled on AS, and (c) after five years, nearly two-thirds of patients benefitted from delaying active treatment by remaining on AS, switching over to WW, or dying of causes other than PCa, while one-third of patients required definitive treatment after five years on AS. Again, many of these conclusions were also observed by others, albeit over shorter study durations.

The incidental discovery of PCa on an otherwise routine TURP (T1a/b) for BPH management is a common and challenging situation that merits further discussion. First, a majority of the landmark studies on AS report data on those who received prostate biopsies (T1c) in response to elevated PSAs or pathological DREs [7,22], but few, if any, studies have addressed the differences in disease progression between T1a/b and T1c tumors. This is all despite the fact that the incidence of incidentally found PCa is relatively high, at between 8 and 15% [23,24,25]

Second, most studies fail to differentiate T1a/b and T1c tumors or have a relatively underrepresented cohort of patients with T1a/b disease [7,22]. Here, we prospectively assessed our T1a/b and T1c subgroups with respect to their reclassification rates, which occurred approximately thrice as quickly in the T1c group (reclassification per 3.6 years) versus the T1a/b group (reclassification per 11.2 years). One other study has reported similarly, noting a doubled reclassification rate after biopsy in T1c patients, though its mean FU time was only 2.4 years [15]. Moreover, the delayed reclassification rate in our T1a/b cohort indicates that such patients (a) live longer prior to developing a definitive treatment requirement, and (b) may be better candidates for WW than definitive active treatment. Both are regarded as favorable outcomes from being on AS.

Third, again, all of our T1a/b patients were incidentally diagnosed with PCa in the setting of bothersome non-neurogenic LUTS from benign prostatic hyperplasia meriting management via TURP. Though speculative, the finding of PCa in the transitional zone (TZ) (TURP resection area) as opposed to the peripheral zone (traditional location for PCa) suggests that TZ PCa tumors may be partly or completely resected during TURP, potentially enabling more durable AS outcomes in this subgroup.

We also assessed differences in disease progression as a function of biopsy modality. Given the breadth of our study duration, we employed both standardized template TRUS biopsies and targeted MRI/TRUS fusion biopsies. While reclassification rates were not significantly different between the two procedures, there was a trend towards a higher reclassification rate in the fusion biopsy group, which is congruent with the prior efforts that demonstrated increased Gleason grade progression with the more sensitive and specific fusion biopsies [26,27,28,29,30].

This said, it remains to be seen if fusion contributes to the overtreatment of otherwise indolent disease, negating the key benefit of AS or on the other hand may allow higher-risk men to enroll in AS and may decrease the intensity of invasive monitoring [31]. As MRI of the prostate certainly adds value to the diagnosis and follow-up and allows for more precise decision making, MRI alone for follow-up is no alternative at the time, as it is shown that progressive or suspicious lesions on mpMRI are not always associated with pathological progression in the biopsy. Both systematic and fusion biopsies are necessary to reliably diagnose progression during AS [29,32,33,34]. As summarized in a recently published systematic review, mpMRI can be used to detect clinically significant prostate cancer in patients on active surveillance, but at the moment, there are no robust data to support the use of mpMRI instead of biopsies [11]. Further investigation and data from prospective studies are needed. For this reason, we plan to add more information on mpMRI and its implementation in the AS protovol to our database in the future.

In a previous observational study we performed in 2012, we noted that 22% of our cohort were ultimately lost to follow-up [35,36]. Subsequently, we sensitized our patients and staff to the issue and routinely conveyed the importance of consistent, regular monitoring. Moreover, our hospital transitioned to a digital database in 2014, facilitating attempts to organize FU for our patients. These two measures alone decreased the LTF rate to 17.5%. Another reason our LTF rate is relatively high compared to other groups is in part because several patients left our practice and pursued outpatient FU with other physicians in the country [7]. Lastly, we also included those who actively declined further examinations and FUs in our LTF rate, which accounts for more than a third of all patient in our cohort. Despite this, we argue that it is this real-world setting that adds value to our understanding of AS implementation.

Limitations of this study include its single-center setting at a mid-size hospital in Switzerland and, consequently, its relatively small sample size. Further, we did not document additional data from the mpMRIs performed during fusion biopsies. We plan on expanding our database to include data such as PI-RADS scores, extraprostatic extension, lymphadenopathy, and perineural invasion. As targeted MRI/TRUS fusion biopsies tend to have a higher reclassification rate, active therapy might have been favored over active surveillance in the recent past, leading to attrition bias. Despite these limitations, our study cohort spans a relevant, transformative period in urology of over two decades, a period of time during which many changes in guidelines and medical technologies took place. We focused on using continuous variables while adding new variables as new technologies presented themselves.

## 5. Conclusions

This prospective, single-center study spans over two decades and reveals that AS remains a safe option for low-risk PCa even in the long-term. Half of AS patients will encounter disease progression and eventually require definitive management. Half of AS patients benefit from delaying active treatment and are spared the negative side effects of active treatment. Men enrolled in AS after TURP demonstrate favorable outcomes, as patients diagnosed by biopsy are reclassified three times sooner than patients diagnosed by TURP. Targeted MRI/TRUS fusion biopsies tend to have a higher reclassification rate and might again lead to over-treatment of low grade prostate cancer. Nonetheless MRI adds value to the diagnosis and follow-up, and allows for more precise decision making.

## Figures and Tables

**Table 1:** Patient characteristics at inclusion.

Variable	Median (IQR) or Number (%)
Age, years	67 (62–71)
PSA, ng/ml	4.8 (3.1–7.5)
PSAD, ng/mL/cm^3^	0.11 (0.08–0.16)
PVol, cm^3^	42.7 (31.7–62)
GS ≤ 6	374 (95.7%)
GS = 7	16 (4.1%)
T1a-b	124 (31.7%)
T1c	267 (68.3%)
FU, months	46 (25–90)

PSA = Prostate specific antigen, PSAD = PSA-Density, PVol = Prostate volume, GS = Gleason score, T1a = incidental histological finding in 5% or less of tissue resected, T1b = incidental histological finding in more than 5% of tissue resected, T1c = Tumor identified by biopsy, FU = Follow-up.

**Table 2:** Patient characteristics at inclusion, T1a/b versus T1c.

Variable	T1a/b, Median (IQR)	T1c, Median (IQR)
Age, years	69 (64–70)	66 (62–72)
PSA, ng/ml	2.4 (1.4–4.6)	5.3 (4–7.8)
PSAD, ng/mL/cm^3^	0.08 (0.04–0.1)	0.12 (0.08–0.17)
PVol, cm^3^	37.7 (28.4–50)	45 (33.4–65)

**Table 3:** Adherence to active surveillance.

Reasons for Discontinuing Active Surveillance	*n*	Percentage of Cohort (391 Patients)
Still on active surveillance	183	46.8%
Disease progression	95	24.3%
Convert to watchful waiting	26	6.6%
Patients choice for active treatment	3	0.8%
Lost to follow-up	68	17.4%
Death	16	4.1%
Total	391	100%

**Table 4:** Treatment types after reclassification.

Treatment Type	*n*	Percentage of All Treatment Types
EBRT	31	30.7%
RP	56	55.4%
Focal therapy	6	5.9%
ADT	3	3.0%
ADT + ERBT	3	3.0%
ADT + CT	1	1.0%
Brachytherapy	1	1.0%
Total	101	100

EBRT = External beam radio therapy, RP = Radical prostatectomy, ADT = Androgen deprivation therapy, CT = Chemotherapy.

## Data Availability

The data presented in this study are available on request from the corresponding author.

## Abbreviations

ADT	Androgen deprivation therapy
AS	Active surveillance
CSS	Cancer specific survival
CT	Chemo-therapy
DRE	Digital rectal examination
EAU	European association of urology
EBRT	External beam radio therapy
FU	Follow-up
GS	Gleason score
LFT	Lost to follow-up
LUTS	Lower urinary tract symptoms
mpMRI	multi-parametric magnetic resonance imaging
OS	Overall survival
PCa	Prostate cancer
PI-RADS	Prostate Imaging Reporting & Data System
PSA	Prostate specific antigen
PSAD	PSA-Density
PVol	Prostate volume
RFS	recurrence-free survival
RP	Radical prostatectomy
TRUS	Transrectal ultrasound
TURP	Transurethral resection of the prostate
TZ	Transitional zone
WW	Watchful waiting
